# Supplementary material for: Evaluation of a Group Therapy for Work-Related Mental Disorders
Source: Int J Environ Res Public Health. 2023 Feb 2;20(3):2673. doi: 10.3390/ijerph20032673 (PMC9915433; doi:10.3390/ijerph20032673)
Supplement: Supplementary file 1 [file ijerph-20-02673-s001.zip › ijerph-2116059-supplementary.pdf]

International Journal of
Environmental Research and Public Health

MDPI

Table S1. Socio-demographic and occupational characteristics of the completer and intention to treat sample and group comparison.

	Completer N = 48	Intention to Treat N = 71	Comparison Completer to ITT p
**Gender, n (%)**			0.47
Women	23 (47.9)	32 (45.1)	
Men	25 (52.1)	39 (54.9)	
**Age, years, n (%)**			0.31
26-40	8 (16.7)	17 (23.9)	
41-50	16 (33.3)	19 (26.8)	
51-60	24 (50)	35 (49.3)	
**Marital status, n (%)**			0.61
Single	11 (22.9)	18 (25.4)	
Married	23 (47.9)	32 (45.1)	
Widowed	-	1 (1.4)	
Divorced	11 (22.9)	16 (22.5)	
Seperated	2 (4.2)	3 (4.2)	
Missing	1 (2.1)	1 (1.4)	
**Children, n (%)**			0.63
Yes	30 (62.5)	43 (60.6)	
No	18 (37.5)	28 (39.4)	
**Migration background, n (%)**			0.73
Yes	5 (10.4)	8 (11.3)	
No	43 (89.6)	63 (88.7)	
**Education, n (%)**			0.06
Primary School	3 (6.3)	9 (12.7)	
Middle School	19 (39.6)	25 (35.2)	
Secondary school	26 (54.2)	37 (52.1)	
**Professional qualification, n (%)**			0.74
Apprenticeship	18 (37.5)	27 (28.0)	
Master (craftsmen)	7 (14.6)	8 (11.3)	
University	19 (39.6)	27 (38.0)	
None	1 (2.1)	1 (1.4)	
Other	3 (6.3)	5 (7.0)	
**Professional group, n (%)**			0.09
Skilled worker	2 (4.2)	6 (8.5)	
Employee with simple tasks	2 (4.2)	4 (5.6)	
Employee with qualified tasks	24 (50.0)	30 (42.3)	
Employee with high qualified tasks/ leadership	14 (29.2)	19 (26.8)	
Employee in extensive leading position	5 (10.4)	8 (11.3)	
Other	1 (2.1)	4 (5.6)	
**Employment status, n (%)**			0.55
Full-time	36 (75.0)	51 (71.8)	
Part-time	12 (25.0)	19 (26.8)	

Table S2. Number of ICD-10 coded mental and behavioral disorders of completer and intention to treat sample.

	Completer Sample N = 48	Intention to Treat Sample N=71
**Number mental and behavioral disorders, n (%)**		
One	32 (66.7)	45 (63.4)
Two	12 (25.0)	16 (22.5)
Three	4 (8.3)	6 (8.5)
Missing	-	4 (5.6)
**Main psychiatric diagnoses, n (%)**		
**Mood affective disorders**	30 (62.5)	40 (56.3)
Mild depressive episode	5 (10.4)	6 (8.5)
Moderate depressive episode	10 (20.8)	15 (21.1)
Recurrent depressive disorder, current episode mild	4 (8.3)	6 (8.5)
Recurrent depressive disorder, current episode moderate	10 (20.8)	12 (16.9)
Recurrent depressive disorder, current episode severe without psychotic symptoms	1 (2.1)	1 (1.4)
**Neurotic, stress-related and somatoform disorders**	13 (56.3)	22 (63.3)
Panic disorder	1 (2.1)	2 (2.8)
Generalized anxiety disorder	1 (2.1)	3 (4.2)
Mixed anxiety and depressive disorder	1 (2.1)	1 (1.4)
Adjustment disorders	5 (10.4)	7 (9.9)
Other reactions to severe stress	2 (4.2)	2 (2.8)
Undifferentiated somatoform disorder	2 (4.2)	3 (4.2)
Somatoform autonomic dysfunction	1 (2.1)	2 (2.8)
Persistent somatoform pain disorder	-	1 (1.4)
**Behavioral syndromes associated with physiological disturbances and physical factors**	3 (6.3)	3 (4.2)
Eating disorder, unspecified	1 (2.1)	1 (1.4)
Psychological and behavioral factors associated with disorders or diseases classified elsewhere	2 (4.2)	2 (2.8)
**Disorders of adult personality and behavior**	2 (4.2)	2 (2.8)
Other specified disorders of adult personality and behavior	2 (4.2)	2 (2.8)
**Total psychiatric diagnoses, n (%)**		
Mood (affective) disorders	39 (60.9)	55 (57.9)
Neurotic, stress-related and somatoform disorders	17 (26.5)	29 30.5)
Behavioral syndromes associated with physiological disturbances and physical factors	4 (6.3)	7 (7.4)
Disorders of adult personality and behavior	4 (6.3)	4 (4.2)

Table S3. Symptoms at admission and discharge.

	T1					
	**Completer Sample (N=48) M(SD)**	**ITT Sample (N=71) M(SD)**	**Completer Women (n=23) M(SD)**	**ITT Women (n=32) M(SD)**	**Completer Men (n=25) M(SD)**	**ITT Men (n=39) M(SD)**
**PHQ-15**	10.81 (5.61)	11.7 (5.8)	12.74 (5.43)	14.47 (5.98)	9.04 (5.27)	9.44 (4.63)
**PHQ-15***	10.73 (5.56)	11.44 (5.56)	12.57 (5.38)	13.91 (5.68)	9.04 (5.27)	7.62 (4.08)
**PHQ-9**	11.06 (6.19)	11.03 (5.4)	12.25 (5.97)	11.88 (5.40)	9.96 (5.50)	10.33 (5.35)

**GAD-7**	9.94 (5.18)	8.55 (4.7)	10.74 (4.85)	9.56 (4.70)	9.20 (5.45)	7.72 (4.66)
**MBI EE**	4.63 (.95)	4.7 (.94)	4.87 (.77)	5.00 (.75)	4.42 (1.07)	4.50 (1.02)
**MBI Z**	3.93 (.99)	4.0 (.99)	4.00 (.86)	4.02 (8.3)	3.86 (1.11)	3.98 (1.11)
**MBI PF**	4.30 (.83)	4.3 (.75)	4.31 (.74)	4.23 (.70)	4.27 (.93)	4.36 (.80)
			**T2**			
	**Completer Sample (N=48) M(SD)**	**ITT Sample (N=71) M(SD)**	**Completer Women (n=23) M(SD)**	**ITT Women (n=32) M(SD)**	**Completer Men (n=25) M(SD)**	**ITT Men (n=39) M(SD)**
**PHQ-15**	9.85 (6.19)	10.99 (6.26)	10.30 (5.80)	12.59 (6.76)	9.44 (6.62)	9.44 (4.63)
**PHQ-15***	9.73 (6.12)	10.76 (6.03)	10.04 (5.67)	12.09 (6.39)	9.44 (6.62)	9.44 (4.63)
**PHQ-9**	8.29 (6.27)	9.15 (5.88)	8.17 (5.75)	8.94 (5.33)	8.40 (6.83)	9.33 (6.35)
**GAD-7**	7.13 (5.69)	7.70 (5.46)	6.17 (4.50)	7.47 (5.16)	8.00 (6.57)	7.9 (5.75)
**MBI EE**	4.05 (1.35)	4.33 (1.28)	4.14 (1.16)	4.48 (1.15)	3.97 (1.52)	4.2 (1.38)
**MBI Z**	3.70 (1.32)	3.85 (1.23)	3.70 (1.19)	3.68 (1.13)	3.72 (1.44)	4.00 (1.30)
**MBI PF**	4.41 (.94)	4.38 (.83)	4.62 (.71)	4.45 (.72)	4.23 (1.09)	4.32 (.92)

PHQ-15 (Patient Health Questionnaire Somatization Module);* without Menstruation Item; PHQ-9 (Patient Health Questionnaire Depression Module); GAD-7 (General Anxiety Disorder Short Scale); Varying sample sizes; significant p-values are marked in bold.

Table S4. Symptom change of the total sample, women, men.

	**Completer Sample T1-T2**			**Completer Women T1-T2**			**Completer Men T1-T2**		
	**T**	**p**	**d**	**T**	**p**	**d**	**T**	**p**	**d**
**PHQ-15**	1.57	0.12	0.23	3.65	**<.001**	**0.76**	-0.43	0.67	-0.09
**PHQ-15***	1.61	0.11	0.23	3.59	**<.05**	**0.75**	-0.43	0.67	-0.09
**PHQ-9**	3.6	**<.001**	**0.53**	4.62	**<.001**	**0.96**	1.32	0.20	0.26
**GAD-7**	3.4	**0.001**	**0.49**	4.36	**<.001**	**0.91**	1.02	0.32	0.20
**MBI EE**	3.8	**<.001**	**0.55**	3.26	**<.05**	**0.68**	2.13	**<0.05**	**0.43**
**MBI C**	1.50	0.14	0.22	1.51	.15	0.32	0.65	0.52	0.13
**MBI PF**	-1.20	0.24	-0.17	-2.70	**<.05**	**-0.56**	0.41	0.68	0.08
	**ITT Sample T1-T2**			**ITT Women T1-T2**			**ITT Men T1-T2**		
	**T**	**p**	**d**	**T**	**p**	**d**	**T**	**p**	**d**
**PHQ-15**	1.64	0.10	0.20	3.21	**<0.05**	**0.57**	-0.39	0.70	-0.06
**PHQ-15***	1.53	0.12	0.19	3.21	**<0.05**	**0.57**	-0.39	0.70	-0.06
**PHQ-9**	3.49	**<0.001**	**0.41**	4.12	**<0.001**	**0.73**	2.54	0.20	0.21
**GAD-7**	1.59	0.12	0.19	2.84	**<0.05**	**0.50**	1.28	0.81	-0.40
**MBI EE**	3.61	**<0.001**	**0.43**	3.03	**<0.05**	**0.54**	0.57	**<0.05**	**0.33**
**MBI C**	.82	0.42	0.10	1.56	0.13	0.28	0.51	0.94	-0.01
**MBI PF**	-1.20	0.24	-0.14	-2.60	**<0.05**	**-0.46**	0.23	0.68	0.07

PHQ-15 (Patient Health Questionnaire Somatization Module);* without Menstruation Item; PHQ-9 (Patient Health Questionnaire Depression Module); GAD-7 (General Anxiety Disorder Short Scale); Varying sample sizes; significant p-values are marked in bold.
